# Effect of DMSO on the Mechanical and Structural Properties of Model and Biological Membranes

**DOI:** 10.1016/j.bpj.2020.05.037

**Published:** 2020-06-15

**Authors:** Beatrice Gironi, Zehra Kahveci, Beth McGill, Bob-Dan Lechner, Stefano Pagliara, Jeremy Metz, Assunta Morresi, Francesca Palombo, Paola Sassi, Peter G. Petrov

**Affiliations:** 1 Dipartimento di Chimica Biologia e Biotecnologie, Università di Perugia, Perugia, Italy; 2 Living Systems Institute and School of Biosciences, University of Exeter, Exeter, United Kingdom; 3 Department of Physics and Astronomy, University of Exeter, Exeter, United Kingdom

## Abstract

Dimethyl sulfoxide (DMSO) is widely used in a number of biological and biotechnological applications, mainly because of its effects on the cell plasma membrane, but the molecular origins of this action are yet to be fully clarified. In this work, we used two- and three-component synthetic membranes (liposomes) and the plasma membrane of human erythrocytes to investigate the effect of DMSO when added to the membrane-solvating environment. Fourier transform infrared spectroscopy and thermal fluctuation spectroscopy revealed significant differences in the response of the two types of liposome systems to DMSO in terms of the bilayer thermotropic behavior, available free volume of the bilayer, its excess surface area, and bending elasticity. DMSO also alters the mechanical properties of the erythrocyte membrane in a concentration-dependent manner and is capable of increasing membrane permeability to ATP at even relatively low concentrations (3% v/v and above). Taken in its entirety, these results show that DMSO is likely to have a differential effect on heterogeneous biological membranes, depending on their local composition and structure, and could affect membrane-hosted biological functions.

## Significance

Dimethyl sulfoxide (DMSO) has found numerous applications in chemistry, biology, and medicine, most notably as an efficient cell cryoprotectant. There is substantial evidence that it affects the organization and properties of bilayer lipid membranes. In this work, we demonstrate that the effect of DMSO on membrane lipid packing, available free volume, and its thermotropic and mechanical properties is not universal but depends on membrane composition and physical state (liquid disordered versus liquid ordered). We also show that DMSO significantly increases the permeability of the human erythrocyte membrane to ATP even at low sulfoxide concentrations, which could be important in the context of cryopreservation of cells.

## Introduction

The plasma membrane is a key constituent of the cell (1), providing the basis for its entire structural integrity but also enabling a large number of cellular functions, including passive and active transport, cell-cell and cell-matrix communication, chemical and mechanical signal transduction, motility, and shape adaptation. The supramolecular organization of the membrane (both lateral and transverse) and its physical properties (viscoelasticity, electrical potentials, etc.) play important roles in controlling and directing biological function (see e.g., (2, 3, 4)) and, if compromised, could lead to impaired function and disease (5, 6, 7, 8). The membrane is the first barrier that natural and exogenous agents encounter in their initial approach to the cell, and the outcome of their interactions with the membrane could be decisive for the fate of the cell, especially in the case of highly reactive compounds ranging from reactive oxidative species to membrane active peptides and toxins. Other compounds commonly used in laboratory or clinical practice could also have far-reaching effects on the plasma membrane organization and physical properties (hence, function), and even though their impact may not be dramatically manifested, they could elicit more subtle, but important, membrane responses on different timescales, leading to modulated or impaired membrane function. A primary example of such a scenario is the modification of the membrane organization and physical properties of stored red blood cells (RBC) (9) and its importance for blood transfusion (10). It is therefore important to understand and quantify the effects that commonly used exogenous species have on the membrane as a basis for clarifying altered function.

In this work, we investigate the effect of dimethyl sulfoxide (DMSO) on synthetic and natural lipid bilayer membranes. DMSO has found numerous uses, primarily as a solvent and a penetration enhancer for various compounds (including drugs) (11,12) but also as an efficient cell cryoprotectant (13,14), typically used at concentrations of 10%. It also exhibits fusogenic activity (15, 16, 17) and is able to induce cell differentiation (18). Although soluble in water, the DMSO molecule is amphiphilic, which explains its affinity toward the plasma membrane. Recent research on the effects of DMSO on lipid membranes has revealed important aspects of this interaction, affecting a range of properties and behaviors of membranes and membrane-bound water. It has been established that DMSO acts in a concentration-dependent manner. Generally, at lower concentrations (up to ∼10 mol%), loosening (permeation, deformability) of the membrane has been observed in fibroblasts (19). Upon an increase in concentration, DMSO enhances membrane permeability both to smaller molecules and ions (water, Ca^2+^) and to larger molecules (Yo-Pro-1 iodide, which is unable to cross the membrane in the absence of DMSO) (19). Experiments have also revealed the effect of DMSO on membrane-bound water. The addition of DMSO causes an increase in the diffusivity of water molecules at the surface of gel phase dipalmitoylphosphatidylcholine (DPPC) bilayers and decreases the range and strength of repulsion between adjacent bilayers (20). The latter agrees well with x-ray and neutron scattering studies reporting decreases in lamellar and hexagonal repeat distances with increased DMSO concentrations (21, 22, 23, 24, 25, 26, 27). These results suggest that DMSO dehydrates the lipid headgroups, thereby effectively “decoupling” water from the membrane (20). Similar effects on surface water diffusivity have been obtained for other membrane compositions, including positively and negatively charged lipid bilayers, irrespectively of the bilayer’s lipid composition and phase (gel or fluid) (27). Physiologically relevant effects in cells have also been reported, e.g., increased neurotransmitter release from cells induced by low concentrations of DMSO (less than 1%) (28).

Molecular dynamics (MD) simulations have been successfully employed to reveal details of DMSO-membrane interactions on atomistic scales inaccessible by experiment and thereby clarify mechanisms behind experimental observations. MD simulations identified three distinct regimes of DMSO action at increasing concentrations in single-component and cholesterol-containing lipid bilayers: membrane loosening, thinning, and increase of membrane hydrophobic core fluidity at low concentrations (up to ∼10 mol%), followed by the formation of pores of increasing stability (for DMSO concentrations up to 40 mol%, although this value depends on the bilayer composition) and, eventually, disintegration of the bilayer at high DMSO concentrations (19,29, 30, 31). These findings could explain the increased membrane permeability of different size molecules observed in experiments. Notably, simulations predicted that DMSO has a marked disordering effect on the membrane, even at a relatively low concentration (up to 10 mol%), manifested by an increase in membrane area (31,32) and in free space in the membrane hydrophobic region (19), effects which we address experimentally in this study. As argued later, again on the basis of MD simulations (33), such structure-altering properties of DMSO could be exploited to facilitate electroporation of lipid membranes. Simulations also predicted significant alterations in membrane elasticity, e.g., 3.7-fold decrease in area compressibility and a fivefold decrease in membrane bending modulus of DPPC bilayers in the presence of 12 mol% DMSO (29).

This study has been motivated by the need to understand and quantify the effects of DMSO on multicomponent lipid membranes, in particular cholesterol-containing lipid bilayers and the importance of the membrane physical state. We address these questions using synthetic membranes with two different compositions, 1-palmitoyl-2-oleoyl-*sn*-glycero-3-phosphocholine (POPC)/egg sphingomyelin (ESM) 1:1 and POPC/ESM/cholesterol 1:1:3, containing the most abundant lipid species of the cell plasma membrane, and interrogate the mechanics and structure of these systems using membrane thermal fluctuation analysis and Fourier transform infrared (FTIR) spectroscopy. We demonstrate that there are marked differences in the response of these two systems to DMSO, with more pronounced effects observed with the two-component bilayers. Because lipid bilayers, such as liposomes, are simple models for the plasma membrane of cells and do not replicate in full important features of cell membranes such as lateral domain organization, interleaflet asymmetry, protein content, membrane skeleton, and glycocalyx etc., we also investigate the effect of DMSO on the membrane of human RBC. We demonstrate that DMSO alters the elasticity of the RBC membrane and enhances membrane permeability to ATP, even at relatively low DMSO concentrations.

## Materials and Methods

### Materials

ESM, POPC, and cholesterol (Chol) were purchased from Avanti Polar Lipids (Alabaster, Alabama) in a powder form and used without further purification. DMSO (purity >99%), tungsten hexacarbonyl ((W(CO)_6_) (purity >99%), sucrose, glucose, Texas Red 1,2-dihexadecanoyl-*sn*-glycero-3-phosphoethanolamine, triethylammonium salt), and Tris buffer were purchased from Sigma-Aldrich (Dorset, UK).

### Preparation of multilamellar vesicles

The multilamellar vesicles (MLVs) were prepared using the lipid film hydration method (34). Lipids were dissolved in chloroform in the appropriate ratio. The solvent was allowed to evaporate under gentle dry nitrogen gas and placed in a vacuum desiccator overnight to remove the organic solvent traces. This resulted in the formation of a thin lipid film on the bottom of the glass vial. Hydration of the lipid film was accomplished by adding either water or water/DMSO solution at DMSO 10 mol% to achieve a total lipid concentration of 100 mg mL^−1^. This ensured that DMSO was homogeneously distributed throughout the MLV with access to all bilayers of the vesicle. The hydration process was conducted at a temperature above the main phase transition of the lipid mixture. The obtained lipid suspension underwent three freeze/thaw cycles by placing the sample vial in liquid nitrogen and in warm water alternately. This procedure resulted in stacked MLVs*.* After preparation, the vesicles were left at 4°C for 1 day before the FTIR measurements. When necessary, W(CO)_6_ dissolved in chloroform was added to the lipid chloroform solution from which the MLVs were formed, ensuring homogeneous distribution of this component throughout the MLV.

### Preparation of giant unilamellar vesicles

Giant unilamellar vesicles (GUVs) were prepared by the electroformation method (35), using the Nanion Vesicle Prep Pro (Nanion Technologies, Munich, Germany). Lipids and lipid mixtures (POPC, POPC/ESM 1:1, and POPC/ESM/cholesterol 1:1:3) were dissolved in chloroform/methanol (2/1, v/v) solution to a final lipid concentration of 10 mM. GUVs were labeled with Texas Red 1,2-dihexadecanoyl-*sn*-glycero-3-phosphoethanolamine for fluorescence microscopy imaging. The dye concentration was kept at less than 1 mol% relative to the total lipid concentration to avoid any undesired effect on the bilayer properties. An aliquot of 5 *μ*L of lipid solution was placed on the conductive side of an indium tin oxide (ITO)-coated glass (VisionTek Systems, Cheshire, UK) within a rubber O-ring and left to dry in a vacuum desiccator for 15 min. After drying, the lipid film within the rubber O-ring was hydrated with 280 *μ*L of 200 mM sucrose solution (water or water/DMSO 5% v/v); a second ITO-coated glass was then placed on top of the O-ring, the conductive side facing the sample solution, and the Nanion Vesicle Prep Pro chamber was closed. Electroformation was then performed with the same parameters for the different lipid mixtures, specifically in three steps at 50°C: 1) the AC voltage was linearly increased from 0 to 1.6 V peak to peak at 10 Hz in 5 min; 2) the voltage was kept constant at 1.6 V peak to peak and 10 Hz for 160 min; and 3) the voltage was decreased linearly to 0 V at 10 Hz in 5 min. After electroformation, GUVs were collected carefully with a micropipette from the ITO slide surface and placed in an Eppendorf tube before use the same day. For fluorescence microscopy measurements, GUVs were carefully added to a microscope chamber (ibidi, Gräfelfing, Germany) filled with 200 mM glucose solution (Tris buffer 10 mM or Tris buffer 10 mM/DMSO 5% v/v) to let the vesicles settle on the bottom of the chamber. The highest concentration of DMSO we managed to achieve for formation of stable GUVs was 5% v/v, and we limited the experiments to this concentration.

### Fluorescence microscopy

Fluorescence images of GUV samples were collected using a 100× oil immersion objective mounted on an Olympus IX73 inverted epifluorescence microscope coupled with a sCMOS camera (Zyla 4.2; Andor Technology, Belfast, UK). Samples were illuminated using a green LED at 10% (pE300white; CoolLED, Andover, UK) and tetramethylrhodamine filter. The acquired microscopy images were used to examine GUV’s morphology. Measurements for each experimental condition were carried out in 15 different areas of the chamber and repeated in at least three independent experiments. A total of at least 100 vesicles were screened for each condition. Lamellarity of vesicles was measured in 15 different areas of the chamber by using a semiautomated algorithm implemented in ImageJ (36). Fluorescence images reported in this study show typical GUV morphology and lamellarity for each experimental condition.

### FTIR spectroscopy of MLVs

Infrared absorption measurements were carried out using a Bruker Tensor 27 FTIR spectrometer (Ettlingen, Germany) with a resolution of 1 cm^−1^ in the 5000–900 cm^−1^ range. For FTIR measurements, 10 *μ*L of lipid suspension of MLVs was sandwiched between two calcium fluoride windows. The temperature was controlled using an FTIR600 Infrared Stage (Linkam Scientific Instruments, Tadworth, UK). The temperature dependence of the FTIR spectra was studied by cooling the sample from room temperature down to a minimum of −60°C at a rate of ∼1°C min^−1^. The sample was thermally equilibrated at −60°C for 30 min and subsequently heated up at a rate of 1°C min^−1^ while acquiring FTIR spectra every 60 s.

### FTIR spectra analysis

Spectral analysis was carried out using OPUS 6.5 Bruker Optics software. As a proxy to membrane fluidity, we analyzed the CH_2_ symmetric stretching, *ν*_s_(CH_2_), band at ∼2850 cm^−1^. The band position was obtained by the “peak picking” routine in OPUS 6.5 software (BrukerOptics), using the “standard mode” evaluation (*x*-coordinate of the relative maximum). By plotting the *ν*_s_(CH_2_)-peak position as a function of temperature, we obtained melting curves for the different samples and determined the temperature of the main phase transition of the membrane, *T*_m_, from the maximum of the first derivative of the melting curves. The temperature dependences of the *ν*_s_(CH_2_)-band position were used to evaluate the parameter $\beta =\left(\partial \nu_{\text{s}}/\partial T\right)/\nu_{\text{s}}$, which shows a very similar temperature behavior to that of the thermal expansion coefficient, α=(∂V/∂T)/V (37), and can therefore be used as a proxy to membrane thermal expansivity. Membrane-solubilized W(CO)_6_ was used to investigate the acyl chain organization and available free volume inside the bilayer. We measured the integrated area of the C≡O antisymmetric stretching, *ν*_as_(CO), of W(CO)_6_ in the range between 1960 and 1990 cm^−1^. Spectra were normalized to the intensity of the *ν*_s_(CH_2_) peak at 25°C to allow for a reliable comparison between different samples.

### Membrane thermal fluctuation analysis of GUVs

**Figure 1:**
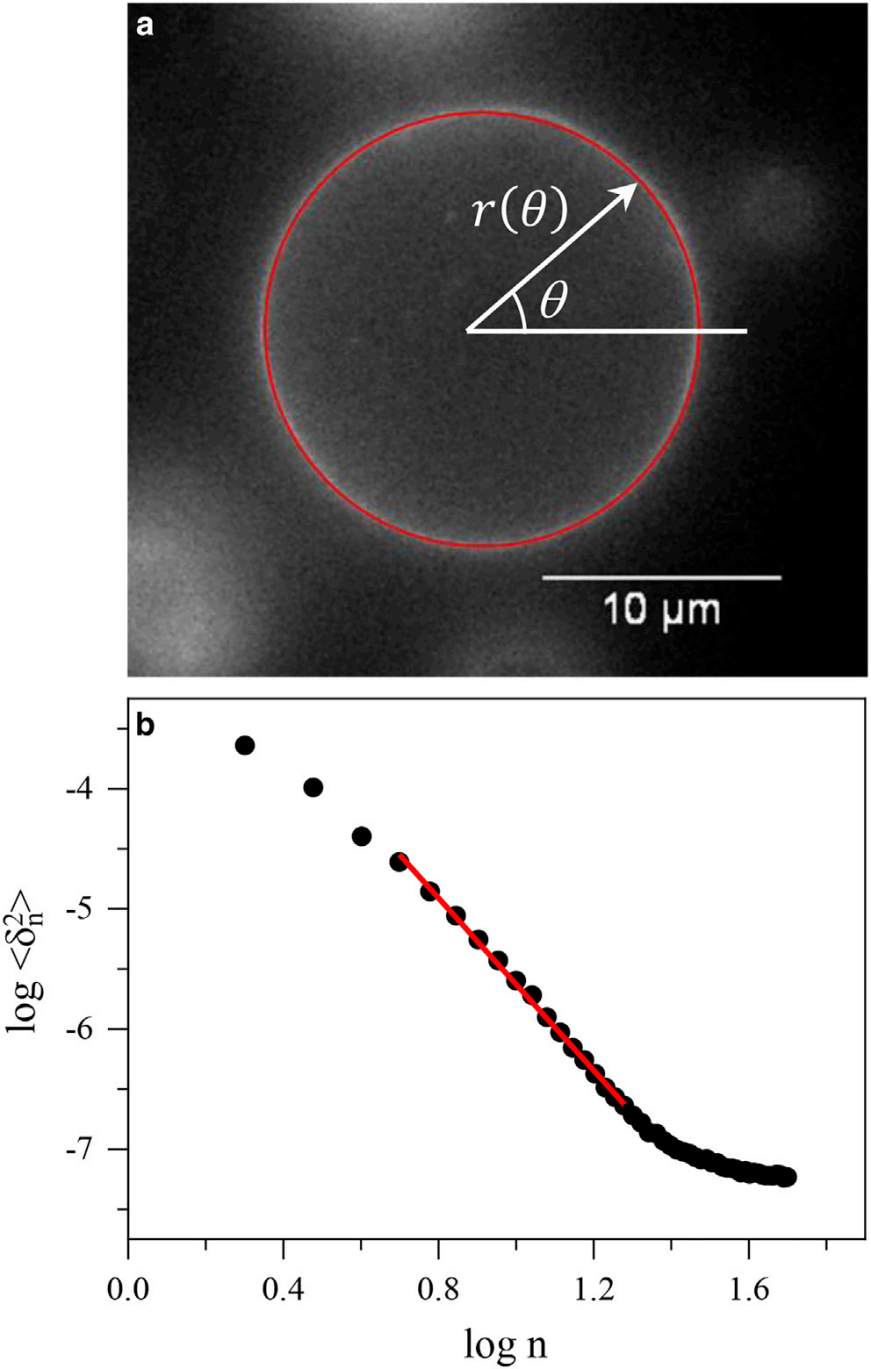
(*a*) Fluorescence microscopy image of a GUV (∼18 *μ*m diameter). The thin red line denotes the traced 2D equatorial contour. *r* and *θ* are the polar coordinates used (with the origin in the contour center). (*b*) Example of a fluctuation spectrum, showing the dependence of the mean-squared fluctuation amplitudes, $\langle \delta_{n}^{2}\rangle$, on the mode number, *n*. The red line is the fit to Eq. 2 for the higher modes. To see this figure in color, go online.

GUV thermal fluctuations were assessed using an inverted fluorescence video microscope (IX73; Olympus, Tokyo, Japan) equipped with 20% green LED light source (pE300white; CoolLED) and tetramethylrhodamine filters. Video sequences with an exposure time of 15 ms and a typical frame rate of 60 fps were collected using a 100× oil immersion objective (IX73; Olympus) and an sCMOS camera (Zyla 4.2; Andor Technology). The slight density mismatch between the inner and outer medium of the GUVs stabilized the vesicles on the bottom of a slide chamber for convenient fluorescence imaging, allowing the observation of membrane fluctuations. All experiments were conducted at room temperature (26°C). Two-dimensional (2D) equatorial contours of quasispherical vesicles were extracted using an algorithm based on the detection of the maximum intensity along the equator of the membrane. A series of contours (between 2000 and 2500) was then analyzed to quantify the thermal fluctuation amplitudes. Each contour was represented by a Fourier series, $r\left(\theta \right)=R\left\{1+\sum_{n}\left[a_{n}\;\cos\left(n\theta \right)+b_{n}\;\sin\left(n\theta \right)\right]\right\}$ (38,39), using polar coordinates (*r, θ*) with the origin at the contour center (see Fig. 1 *a*).

Contour mean-squared fluctuation amplitudes around the mean contour shape were quantified by using the Fourier amplitudes, $\langle \delta_{n}^{2}\rangle =\left[\langle a_{n}^{2}\rangle -{\langle a_{n}\rangle }^{2}\right]+\left[\langle b_{n}^{2}\rangle -{\langle b_{n}\rangle }^{2}\right]$ (39,40), and the fluctuation spectrum was represented as $\langle \delta_{n}^{2}\rangle$ versus mode number *n*, as shown in Fig. 1 *b*. We were able to record successfully modes up to *n* ≈ 18 before limitations in the optical resolution and integration times start affecting higher modes (see the high-mode tail in Fig. 1 *b*). As discussed in details by Pécréaux et al. (38), the contour fluctuation spectrum in the planar membrane approximation can be written as follows:

(1) $$\langle \delta_{n}^{2}\rangle =\frac{1}{2\pi }\frac{1}{\tilde{\kappa }\tilde{\sigma }}\left[\frac{1}{n}-{\left(2\tilde{\sigma }+n^{2}\right)}^{-1/2}\right],$$

where $\tilde{\kappa }=\left(\kappa /k_{\text{B}}T\right)$ is the dimensionless bending modulus, $\tilde{\sigma }=\sigma {\langle R\rangle }^{2}/2\kappa$ is the dimensionless membrane tension (the corresponding dimensional quantities are *κ* and *σ*, respectively), and 〈R〉 is the mean contour radius. In the case of a quasispherical vesicle, the first few (long wavelength) modes are affected by the overall curvature of the membrane and membrane tension *σ* (38); however, higher modes (*n* ≥ 5) can be used to determine the membrane bending elastic modulus *κ*. For the higher (short-wavelength) modes, we obtain

(2) $$\langle \delta_{n}^{2}\rangle \approx \frac{1}{2\pi \tilde{\kappa }}n^{-3}$$

Eq. 2 was used to fit modes in the range *n* = 5–18 of each fluctuation spectrum, hence evaluating *κ*, as shown in Fig. 1 *b*.

### Blood sample acquisition

Blood samples were acquired using a finger prick or venepuncture from healthy individuals in the age group 20–30 years old. Blood was donated voluntarily after giving informed consent and in accordance with the UK ethics regulations (UE eEthics: eEMPS000064). Samples were prepared immediately before the experiments and, if necessary, stored at 4°C and kept no longer than 1 day.

### Membrane thermal fluctuation analysis of RBC

For single RBC thermal fluctuation analysis, 1–3 *μ*L blood samples were obtained via finger prick and immediately diluted in 1 mL phosphate buffer (PBS, 10 mM, pH = 7.4, containing 137 mM NaCl, 2.7 mM KCl, and 1 g L^−1^ bovine serum albumin; Sigma-Aldrich). 20 *μ*L of the RBC suspension were introduced in a thin microscope slide glass chamber (Thermo Fisher Scientific, Loughborough, UK) with a total volume of ∼220 *μ*L. After allowing the RBCs to settle at the bottom of the chamber for 10 min at room temperature, 200 *μ*L of pure buffer were added. Video sequences of selected single RBCs were acquired with a 63× long working distance objective (PL FLUOTAR; Leica, Wetzlar, Germany) using a Leica DMLFS microscope equipped with an MS2000 *xy* stage (Micrasys, Herborn, Germany) in phase contrast mode. Typically, 2000 contours were recorded at 10-ms exposure time with a QImaging QIClick CCD camera (QImaging, Marlow, UK) mounted on the microscope. The sample chamber allowed for the exchange of the buffer solution surrounding the RBCs. After recording the untreated cells, the buffer was exchanged with one containing DMSO (1–10% v/v). In total, 2 mL of the newly introduced buffer was flushed through the chamber over ∼5 min to ensure full buffer exchange. During this procedure, most of the RBCs remain in the field of view because of slight adhesion to the bottom of the glass chamber, which allows the same cells to be imaged after the buffer is exchanged. Subsequently, video footage of the same RBCs was acquired in regular time intervals of 10 min for up to 90 min. For statistical analysis, several cells per sample and several different samples were measured.

RBC thermal fluctuation spectra were obtained in a similar way as for GUVs, as described above. RBC thermal fluctuations are affected by the presence of a 2D membrane protein skeleton positioned underneath the lipid bilayer and anchored to it through junctional complexes (41). Therefore, we used the following expression for the contour fluctuation spectrum in the planar membrane approximation (see (39) for details):

(3) $$\langle \delta_{n}^{2}\rangle =\frac{1}{2\pi }\frac{1}{\tilde{\kappa }\sqrt{{\tilde{\sigma }}^{2}-\tilde{\gamma }}}\left[{\left(\tilde{\sigma }+n^{2}-\sqrt{{\tilde{\sigma }}^{2}-\tilde{\gamma }}\right)}^{-\frac{1}{2}}-{\left(\tilde{\sigma }+n^{2}+\sqrt{{\tilde{\sigma }}^{2}-\tilde{\gamma }}\right)}^{-\frac{1}{2}}\right],$$

where $\tilde{\gamma }=\gamma {\langle R\rangle }^{4}/\kappa$ is the dimensionless strength of the confinement potential (the corresponding dimensional quantity is *γ*), a parameter accounting for the interaction between the lipid bilayer and the membrane skeleton (41). The bending modulus, *κ*, of the erythrocyte membrane can thus be obtained by fitting this equation to the experimentally obtained spectra (39).

### Erythrocyte ATP release assays

For a quantitative determination of extracellular ATP levels, whole blood samples from venepuncture were diluted in DX-PBS buffer (Sigma-Aldrich) containing 1 g L^−1^ bovine serum albumin and 169–200 g L^−1^ dextran (average molar mass of 250 kg mol^−1^; Acros Organics, Geel, Belgium) to give 1% hematocrit. The ATP level in the sample was determined via a luciferase/luciferin (rL/L) luminescence assay using the ENLITEN kit (Promega, Southampton, UK). For each experiment using this assay, 100 *μ*L of the luciferase/luciferin reagent were added to 100 *μ*L of the diluted blood sample, and the luminescence was detected at room temperature using a luminometer (GloMax 20/20; Promega). To quantify the ATP molar concentrations in blood, calibration curves with ATP standard solutions (provided as a part of the ENLITEN kit) in the range of 10^−7^–10^−10^ M were recorded. Diluted blood samples were incubated with 1–10% v/v DMSO in DX-PBS buffer for 30 min at room temperature, and subsequently, the ATP concentration was measured using the luminescence assay. For statistical analysis, samples with the same DMSO concentration were measured five times under the same conditions.

## Results and Discussion

### Effect of DMSO on structural properties of MLV membranes consisting of binary and ternary lipid mixtures

We investigated lipid bilayer membranes (MLV) of two different compositions: binary mixtures (POPC/ESM 1:1) and ternary mixtures (POPC/ESM/cholesterol 1:1:3). A previous study of similar systems (POPC/palmitoylsphingomyelin (PSM) and POPC/PSM/cholesterol) has reported details of their phase behavior at different compositions and temperature (42). Although one of the lipid components, PSM, is slightly different from ESM used in this study (PSM has a 16:0 saturated acyl chain, whereas ESM contains a majority 86% of the same chain, 6% 18:0, 3% 22:0, and 3% 24:1 chains), the phase diagrams reported in that work can be used as a guide for the bilayer phase of the binary and ternary membranes. Binary POPC/PSM 1:1 MLVs at room temperature are reported to be in a phase-separated state between a liquid disordered (L_d_) and a gel phase (more precisely, the sphingomyelin-enriched SO_2_ phase, one of the two solid-ordered phases observed for this system) (42). Upon an increase in temperature, the membrane undergoes a transition to a uniform liquid disordered phase at around 32°C (42). Our binary system (POPC/ESM 1:1) undergoes a similar transition at 27°C (see below). Ternary POPC/PSM/cholesterol 1:1:3 MLVs are reported to be in the liquid-ordered (L_o_) phase at room temperature.

**Figure 2:**
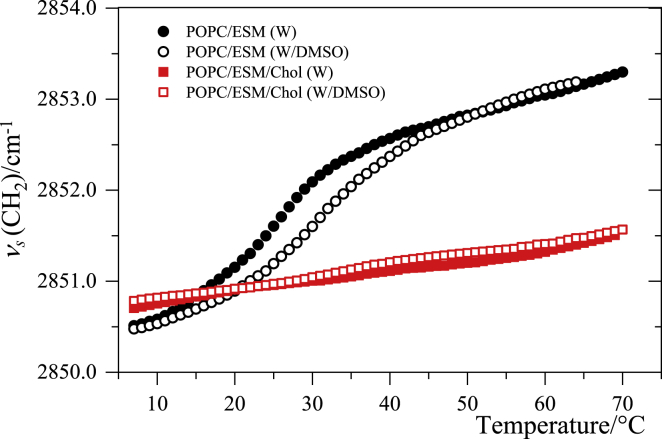
Heating curves showing the temperature dependences of the *ν*_s_(CH_2_) band peak position for the binary (POPC/ESM 1:1) and ternary (POPC/ESM/Chol 1:1:3) MLVs in water (W) and water/DMSO (W/DMSO) solution (10 mol%). Band position is evaluated with ±0.1 cm^−1^ standard error. To see this figure in color, go online.

Fig. 2 shows the temperature trends for the CH_2_ symmetric stretching, *ν*_s_(CH_2_), band at ∼2850 cm^−1^. We used this measurement to obtain “melting curves” and to monitor the bilayer phase transition between 5 and 70°C. Main transition temperatures were obtained from the maximum of the first derivative curves and listed in Table 1. The binary POPC/ESM system in water shows a transition at 27°C, which appears to be associated with the melting of the ESM-enriched gel domains (see also Fig. 2 in (42)). In the presence of DMSO, this melting transition is widened and upshifted to 33°C, increasing the temperature stability of the gel phase in the phase-separated membrane (Table 1). A similar effect on the thermotropic behavior of single lipid systems (phosphatidylcholines) has been observed before (21,43, 44, 45).

**Table 1:** Transition Temperatures

Composition	*x*_DMSO_	T_m_/°C
POPC/ESM 1:1	0	27
POPC/ESM 1:1	0.1	33
POPC/ESM/cholesterol 1:1:3	0	–
POPC/ESM/cholesterol 1:1:3	0.1	–

The thermotropic behavior of the ternary system, POPC/ESM/Chol, is different from that of the binary system (Fig. 2). The presence of a high percentage of cholesterol leads to the formation of a single phase, most probably liquid ordered, L_o_ (42), as the position of the *ν*_s_(CH_2_) band, 2850 cm^−1^, suggests ordered acyl chains and hence a rigid membrane (46, 47, 48). This phase appears to be stable over the entire temperature range investigated. Because of this single phase structure, the presence of DMSO does not affect the membrane thermotropic behavior, in contrast to the behavior observed for the binary system, which is forced across a transition boundary.

**Figure 3:**
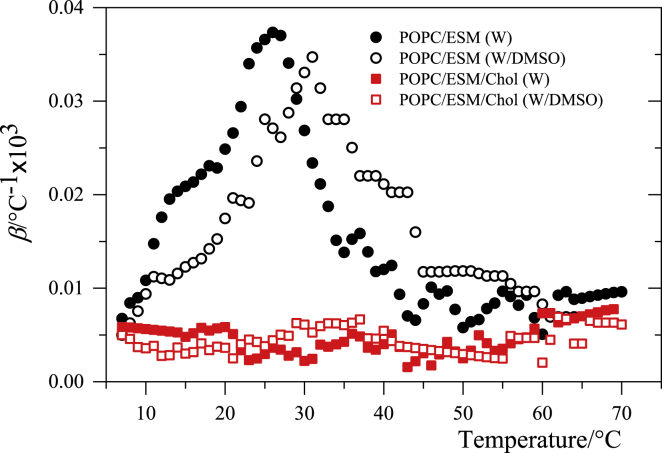
Temperature trend of the parameter $\beta =\left(\partial \nu_{\text{s}}/\partial T\right)/\nu_{\text{s}}$ obtained from the data in Fig. 2. *β* has a temperature dependence similar to that of the thermal expansion coefficient, α=(∂V/∂T)/V (37). To see this figure in color, go online.

The temperature trends based on the *ν*_s_(CH_2_) mode (Fig. 2) allow evaluation of the parameter $\beta =\left(\partial \nu_{\text{s}}/\partial T\right)/\nu_{\text{s}}$, which behaves very similarly to the thermal expansion coefficient α=(∂V/∂T)/V (37,45). The results for both binary and ternary systems are shown in Fig. 3.

An inspection of Fig. 3 shows large values of *β* in the melting range of the binary system. This is probably related to the maximum amplitude of conformational fluctuations that the system undertakes at these temperatures because some segments pass from *trans* to *gauche* conformation and back. On the contrary, at temperatures below 10 and above 45 in which the gel and the liquid phase are respectively stable, the structure of the acyl chain is subject to small conformational fluctuations, and the temperature change has only minor effects. Although DMSO has practically no effect on the magnitude of *β*, its temperature trend is upshifted, indicating a lag in conformational fluctuations in the presence of the sulfoxide.

The ternary system exhibits a low value of *β*, which is essentially constant over the entire temperature range, probably because of the condensing effect of cholesterol in the L_o_ phase (49). The presence of DMSO has no effect on the parameter *β* for the ternary system.

### DMSO and membrane lipid organization

**Figure 4:**
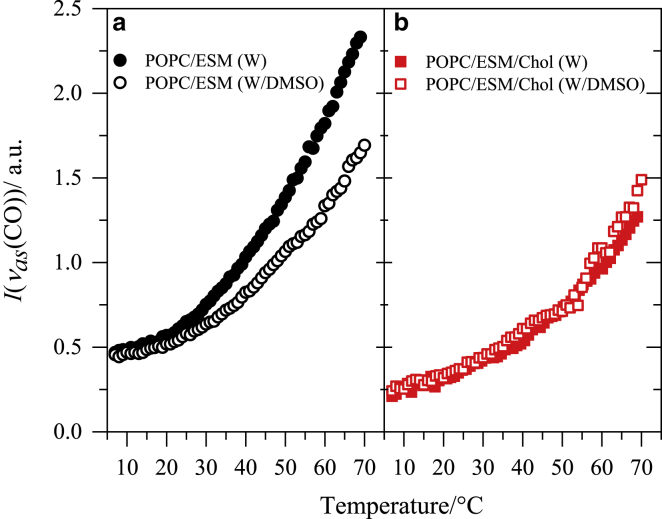
Integrated area of the *ν*_as_(CO) band of W(CO)_6_ in the 1960–1990 cm^−1^ region for (*a*) POPC/ESM (1:1) and (*b*) POPC/ESM/Chol (1:1:3) bilayers in water and water/DMSO (10 mol%) as a function of temperature. To see this figure in color, go online.

Membrane-solubilized W(CO)_6_ was used here as a probe to investigate changes in acyl chain configuration and transversal packing of lipid bilayer membranes. It has been shown that the integrated intensity of the FTIR band corresponding to the C≡O antisymmetric stretching of W(CO)_6_ in the 1960–1990 cm^−1^ range is proportional to the compound concentration within the bilayer’s hydrophobic core (50). Fig. 4 shows this signal-integrated intensity, *I*(*ν*_as_(CO)), as a function of temperature for both the binary (Fig. 4 *a*) and the ternary (Fig. 4 *b*) systems in water and water/DMSO. For the binary system, the W(CO)_6_ solubility gradually increases with increasing temperature. Adding DMSO has the effect of reducing the probe solubility within the bilayer’s hydrophobic core. This result is somewhat surprising because MD simulations performed on DOPC/Chol bilayers (19) have predicted an increase in both membrane area and free space inside the hydrophobic core of the membrane because of the presence of DMSO, which should lead to the opposite effect. On the other hand, results of simulations of pure DPPC bilayers have shown that DMSO, along with area increase, also causes thinning of the membrane (30,31). The latter may explain the experimental observation, provided the membrane thinning is not overcompensated by other structural alterations.

The ternary lipid bilayers are less capable of accommodating W(CO)_6_ than the binary system (Fig. 4 *b*). Also, in contrast to the binary system, DMSO has no discernible effect on the concentration of W(CO)_6_ in the bilayer. This is probably due to the tightly packed arrangement of lipid acyl chains and cholesterol in the L_o_ phase.

**Figure 5:**
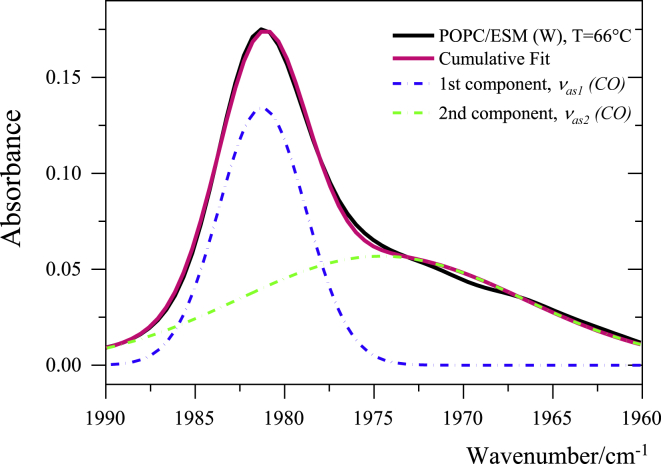
A typical fit of the *ν*_as_(CO) profiles of W(CO)_6_ dissolved in lipid bilayers (POPC/ESM 1:1) in the 1990–1960 cm^−1^ spectral range. The black line denotes the experimental spectrum, whereas the dashed blue and green lines denote the two components, *ν*_as1_(CO) and *ν*_as2_(CO), respectively (the *red line* is the sum of the two). To see this figure in color, go online.

The C≡O antisymmetric stretching band of the membrane-solubilized W(CO)_6_ presents two contributions, at 1981 cm^−1^ (*ν*_as1_(CO)) and 1975 cm^*−*1^ (*ν*_as2_(CO)). The first vibrational mode, *ν*_as1_(CO), is attributed to W(CO)_6_ molecules solubilized close to the center of the membrane, i.e., deep inside the hydrophobic core of the membrane, whereas *ν*_as2_(CO) arises from W(CO)_6_ in proximity to the ester groups in the interfacial region of the bilayer (51,52). The first component, *ν*_as1_(CO), is narrower because of the fact that the probe is surrounded by long hydrocarbon chains that provide a homogeneous environment. The same spectral features have been observed when W(CO)_6_ is solubilized in hexadecane or dodecane. The wider band at 1975 cm^−1^, *ν*_as2_(CO), arises from probe molecules solubilized in the interfacial region of the membrane (near the ester moiety of the lipid) in which the probe experiences less homogeneous environment because of the presence of nearby ester groups. A similar profile has been observed when the probe is solubilized in bis(2-ethylhexyl) succinate or 2-methoxyethylacetate. Fig. 5 shows a typical example of the C≡O peak in the 1990–1960 cm^−1^ spectral range. The peak was reproduced using a curve-fit analysis with two Gaussian functions; the fraction of integrated intensity related to the *ν*_as1_(CO) peak, *f*_1_(CO) = *I*(*ν*_as1_)/(*I*(*ν*_as1_) + *I*(*ν*_as2_)), can serve as a proxy for the amount of W(CO)_6_ “buried” deep inside the hydrophobic core (near the center of the membrane).

**Figure 6:**
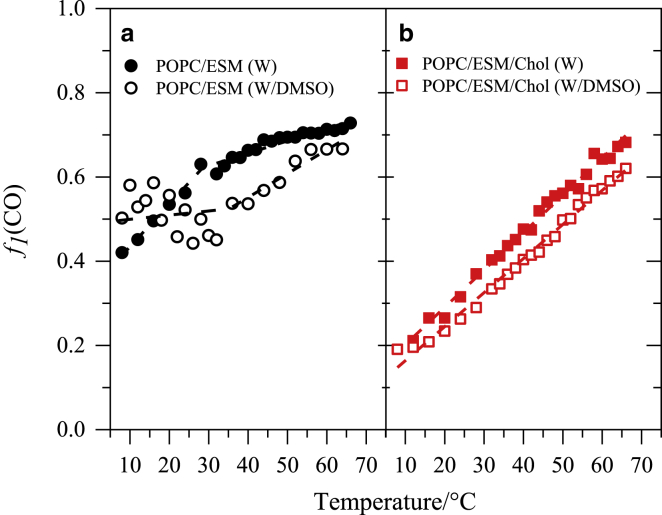
Temperature trends of *f*_1_(CO) for (*a*) POPC/ESM (1:1) vesicles in water and water/DMSO (10 mol%) and (*b*) POPC/ESM/Cholesterol (1:1:3) vesicles in water and water/DMSO (10 mol%). To see this figure in color, go online.

Fig. 6 shows the temperature dependences of the fractional intensity *f*_1_(CO) for the investigated systems (in water and in water/DMSO). *f*_1_(CO) shows distinct features in the behavior of the two lipid systems and the effect of DMSO.

For the binary system in water (Fig. 6 *a*), *f*_1_(CO) increases with increasing temperature, and two different regimes can be identified below and above 27°C, related to the two different phase states of the bilayer membrane. This suggests that an increase in temperature leads to the elevation in the fraction of W(CO)_6_ molecules solubilized in the most hydrophobic region of the bilayer. The presence of DMSO modifies this trend: for lower temperatures (up to the melting temperature, ∼33°C), *f*_1_(CO) remains approximately constant, whereas at higher temperatures, this parameter increases but at a higher rate than in pure water (Fig. 6 *a*). Nevertheless, the value of *f*_1_(CO) in the presence of DMSO remains lower than that for water for almost the whole temperature range (except for low temperatures below ∼15°C), suggesting that the relative amount of W(CO)_6_ accommodated deep into the hydrophobic core of the bilayer is lower than in the absence of DMSO. DMSO may cause a reduction of the available volume close to the center of the membrane, e.g., by causing membrane thinning (30,31). This would explain our previous observation (Fig. 4) of the total amount of W(CO)_6_ incorporated in the membrane.

For the ternary system (Fig. 6 *b*), *f*_1_(CO) increases with a similar gradient for the two solvent compositions, which reflects the lack of any phase transitions in this system. DMSO has a similar effect on the ternary and binary systems: in both cases, the fraction of the probe solubilized in the deep hydrophobic region (i.e., close to the center) of the membrane decreases. In light of Fig. 4 *b*, which suggests that the total amount of W(CO)_6_ in the membrane remains unchanged by DMSO, this result means that DMSO causes a redistribution of W(CO)_6_ in the ternary bilayers from the center of the membrane to the regions adjacent to the headgroups.

### Effect of externally added DMSO on GUV morphology

**Figure 7:**
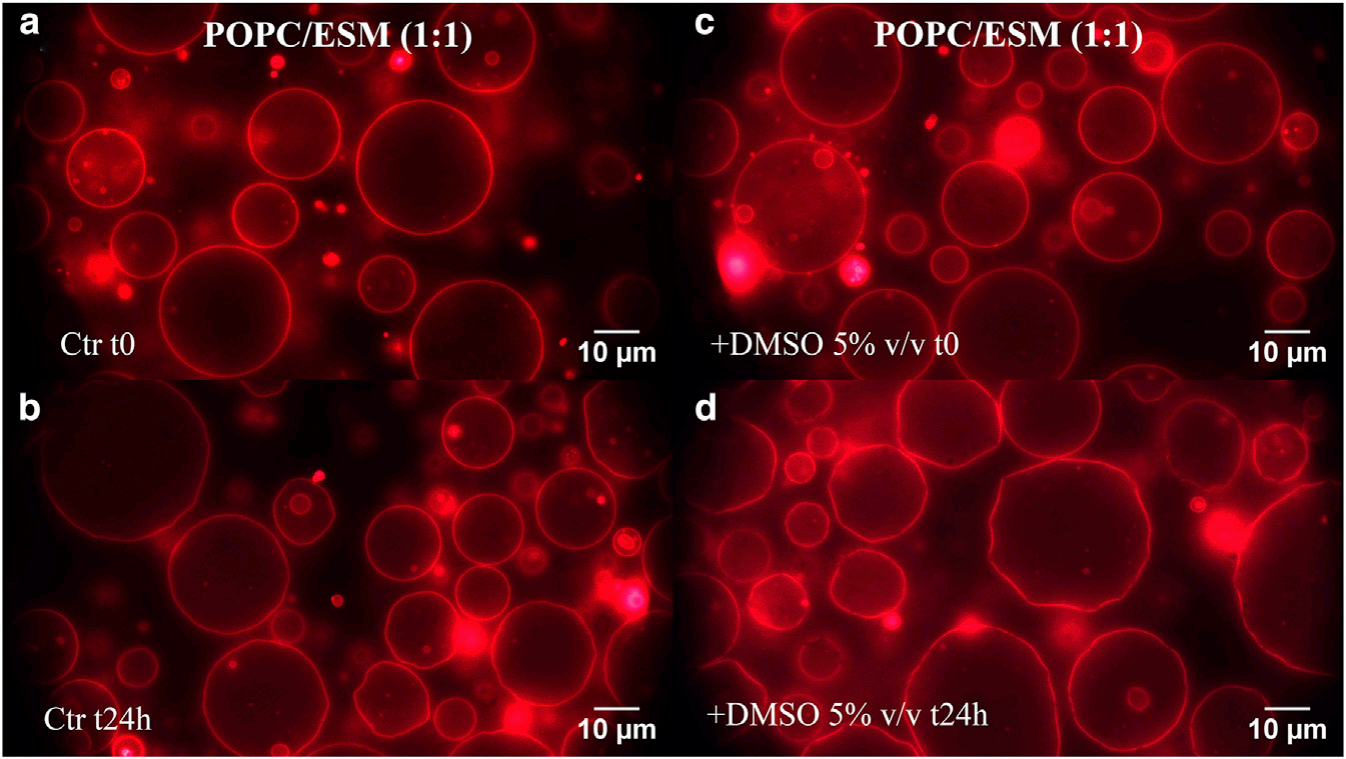
Representative fluorescence microscopy images of POPC/ESM 1:1 GUVs acquired (*a*) immediately after preparation (*t* = 0) and (*b*) after 24 h in water as a control. Images acquired of the binary system with added DMSO (5% v/v) at (*c*) *t* = 0 and (*d*) after 24-h incubation. Membrane undulations are clearly visible in the latter panel. More than 100 vesicles were investigated for each sample. To see this figure in color, go online.

**Figure 8:**
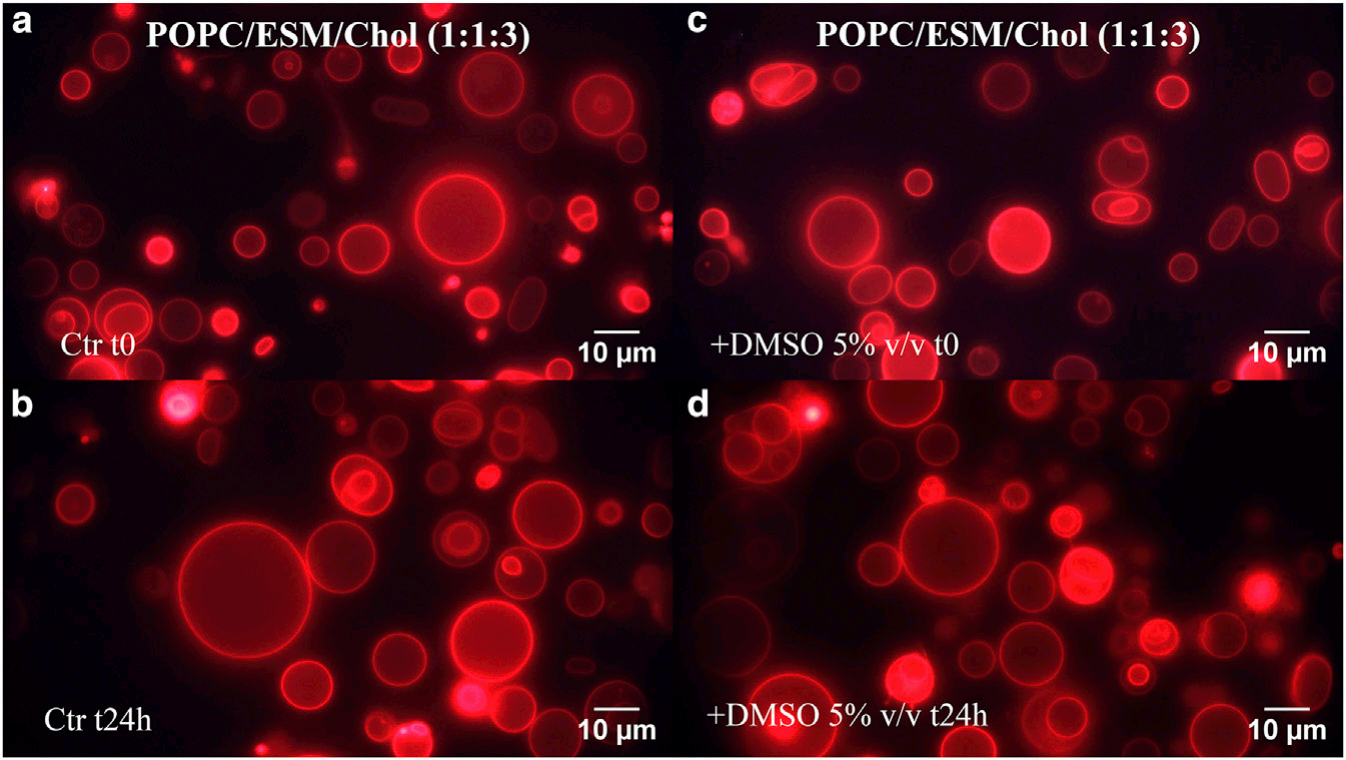
Fluorescence microscopy images of POPC/ESM/cholesterol (1:1:3) GUVs acquired (*a*) immediately after preparation (*t* = 0) and (*b*) after 24 h in water as a control. Images acquired of the ternary system with added DMSO (5% v/v) at (*c*) *t* = 0 and (*d*) after 24-h incubation. More than 100 vesicles were investigated for each sample. To see this figure in color, go online.

Fluorescently labeled GUVs were imaged before and after the addition of DMSO to qualitatively evaluate its effect on the morphology of the vesicles. Fig. 7 (binary system) and Fig. 8 (ternary system) show representative images of GUVs with and without 5% v/v DMSO at different incubation times (0 and 24 h). For the binary mixture (Fig. 7), the majority of the vesicles appear as unilamellar and spherical. After 24 h of incubation with 5% v/v DMSO (Fig. 7 *d*), the vesicles are still unilamellar (judging by the fluorescence intensity of the membrane); however, many appear to have lost their tension and exhibit undulating membranes. This is a clear sign of an increase in the total surface area of the vesicle. The addition of lower DMSO concentrations (<5% v/v) did not alter the GUV morphology (data not shown).

The morphology of the ternary GUVs (Fig. 8) is somewhat different from that of the binary systems; vesicles appear to be more heterogeneous, and some are multilamellar. The addition of DMSO does not appear to have any effect in this case (Fig. 8 *d*).

### Bending rigidity of the GUV membrane

This set of experiments was designed to quantify the effect of DMSO on the membrane’s bending modulus, one of the key mechanical parameters of soft membranes. 5% v/v DMSO was added during GUV formation (as for the morphology studies). The membrane’s bending rigidity was measured using thermal fluctuation spectroscopy, as outlined in the Materials and Methods. All experiments were carried out at room temperature, ∼26°C.

Table 2 shows the mean and the SD of the bending moduli obtained for vesicles of different compositions (POPC, POPC/ESM 1:1, POPC/ESM/Chol 1:1:3, with and without DMSO).

**Table 2:** Membrane Bending Elastic Moduli (in Units of *k*_*B*_T) for GUVs of Different Composition (*n* Is the Number of GUVs Analyzed)

Composition	% v/v DMSO	*n*	*κ*/*k*_B_*T*
POPC	0	4	33 ± 15
POPC	5	3	27 ± 14
POPC/ESM 1:1	0	4	44 ± 18
POPC/ESM 1:1	5	7	40 ± 9
POPC/ESM/cholesterol 1:1:3	0	4	170 ± 30
POPC/ESM/cholesterol 1:1:3	5	6	258 ± 14

The bending moduli obtained for POPC and POPC/ESM GUVs, a few tens of *k*_B_*T*, show the typical values for bilayers in the liquid disordered (L_d_) state (53, 54, 55). For the POPC/ESM vesicles, the value of the bending modulus probably reflects the bending rigidity of the prevalent L_d_ phase. For these two systems, no effect of DMSO on *κ* was detected beyond the experimental uncertainty. If any effect is present, it must be relatively small (see the next section).

The ternary system, POPC/ESM/cholesterol, shows significantly higher values of *κ*. The high values of *κ* reflect the mechanical properties of the L_o_ phase. Similar values for membranes in the L_o_ phase have been reported previously (55, 56, 57). Small variations in lipid composition in different vesicles during electroformation may explain the larger spread in the values for the bending rigidity for the ternary system in water, especially if the vesicles are close to a phase transition boundary (e.g., with L_d_), as has already been observed for the L_o_ phase (55). Table 2 also shows an apparent increase in membrane bending rigidity for ternary GUVs in the presence of DMSO, which again indicates a differential effect of DMSO. In the case of POPC and POPC/ESM GUVs, DMSO does not alter the membrane bending elasticity at the used concentration, whereas it does appear to stiffen the membrane of POPC/ESM/Chol GUVs*.*

### Bending rigidity of the RBC membrane

The RBC offers an excellent system to study the effects of DMSO on a natural cell plasma membrane, which, because of the similar composition and organization, is representative of the membranes of other mammalian cell types. In this section, we present the results of our investigation of the effect of DMSO on the erythrocyte membrane elastic properties. We recorded the response of individual cells by analyzing the thermal fluctuation spectrum of each RBC before and after the addition of DMSO at regular intervals over a period of 90 min. Three different concentrations of DMSO were examined: 1, 5, and 10% v/v.

**Figure 9:**
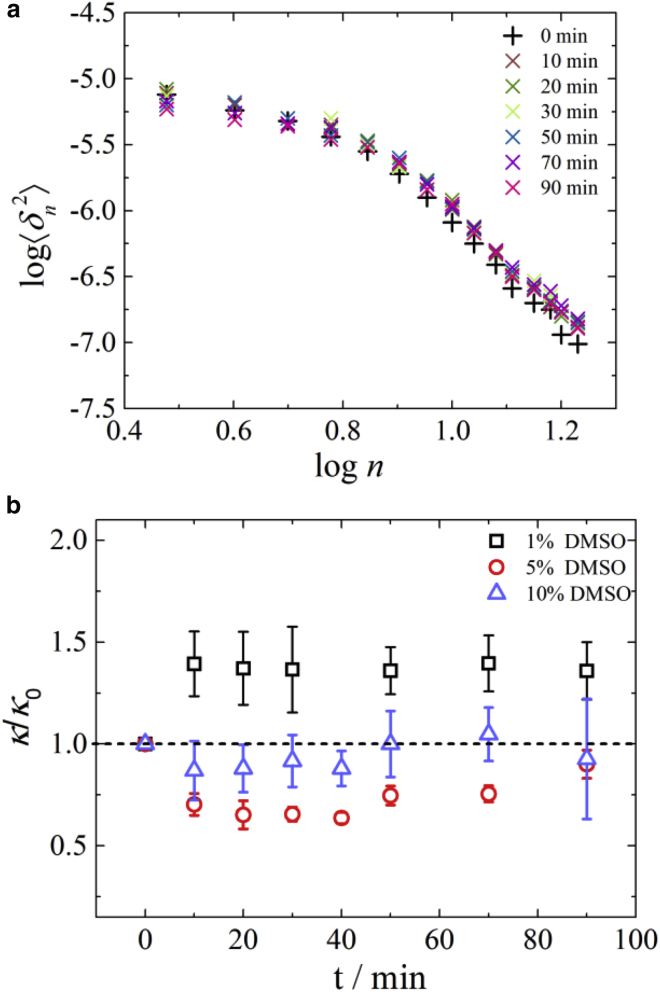
(*a*) Typical evolution in the thermal fluctuation spectrum of an individual RBC over time upon the addition of 5% v/v DMSO. (*b*) Time dependence of the sample averaged bending modulus of RBCs for three DMSO concentrations, 1% v/v (*black squares*). 5% v/v (*red circles*), and 10% v/v DMSO (*blue triangles*). The values of *κ* are normalized to the corresponding bending modulus, *κ*_0_, for each individual cell before the addition of DMSO. Averaged seven cells for 1% v/v DMSO, five cells for 5% v/v DMSO, and seven cells for 10% v/v DMSO. The error bars represent the standard error. To see this figure in color, go online.

Fig. 9 *a* shows the evolution of the thermal fluctuation spectrum of a single cell over time after the addition of DMSO. Qualitatively, changes in the first few (long wavelength) modes of the spectrum indicate changes in the membrane shear modulus (i.e., mechanically significant modifications of the membrane protein skeleton), whereas systematic shifts in the higher (short wavelength) modes can be interpreted as changes in the membrane bending rigidity (attributed to the lipid bilayer) (39).

For example, the higher modes (*n* > 7) in Fig. 9 *a* appear to upshift after the addition of DMSO, i.e., the membrane softens to bending. Quantitative measurement of this effect can be obtained by fitting the experimental spectrum to Eq. 3 to evaluate the bending modulus (Fig. 9 *b*). Note that shear moduli obtained from this model (data not shown) do not change significantly as a result of treatment with DMSO at these concentrations.

Fig. 9 *b* shows the results for all the cells assessed at the three DMSO concentrations. Adding DMSO at 1% v/v causes an increase in the bending modulus by ∼37%, which remains constant over the time period investigated. At 5% v/v DMSO, the trend is reversed as DMSO causes softening of the membrane in terms of bending. In this case, the change in the bending rigidity is not constant in time, and for longer times (90 min), it tends to recover the value for the untreated cells. Membrane softening at this concentration agrees well with findings from a previous experimental study reporting softening of the RBC membrane at higher DMSO concentrations (58) and MD simulations (29), reporting a similar effect; however, to the best of our knowledge, this is the first time that an increase in membrane bending rigidity has been reported for DMSO concentrations as small as 1% v/v. At 10% v/v DMSO, we observe a weaker effect on the membrane bending rigidity, and the bending modulus fully recovers the value for the untreated cells by 50 min. This suggests that DMSO has only a weak effect on membrane mechanics at concentrations typically used for cryopreservation.

### Effect of DMSO on RBC membrane’s permeability to ATP

In this section, we examine whether DMSO can enhance the permeability of the RBC membrane to ATP, in light of the biological importance of this molecule, a problem not addressed before. RBCs are capable of producing ATP by glycolysis via the Embden-Meyerhof pathway (59). To quantify the release of ATP from the RBC interior, fresh whole blood samples were divided into aliquots. One of them, suitably diluted in buffer, was used to measure the extracellular ATP concentration with no DMSO added, as described in the Materials and Methods. The rest of the aliquots were incubated with buffers containing varying concentrations of DMSO (1, 3, 5, 7, and 10% v/v), and the ATP concentration was measured for each sample after 30-min incubation using the same method.

**Figure 10:**
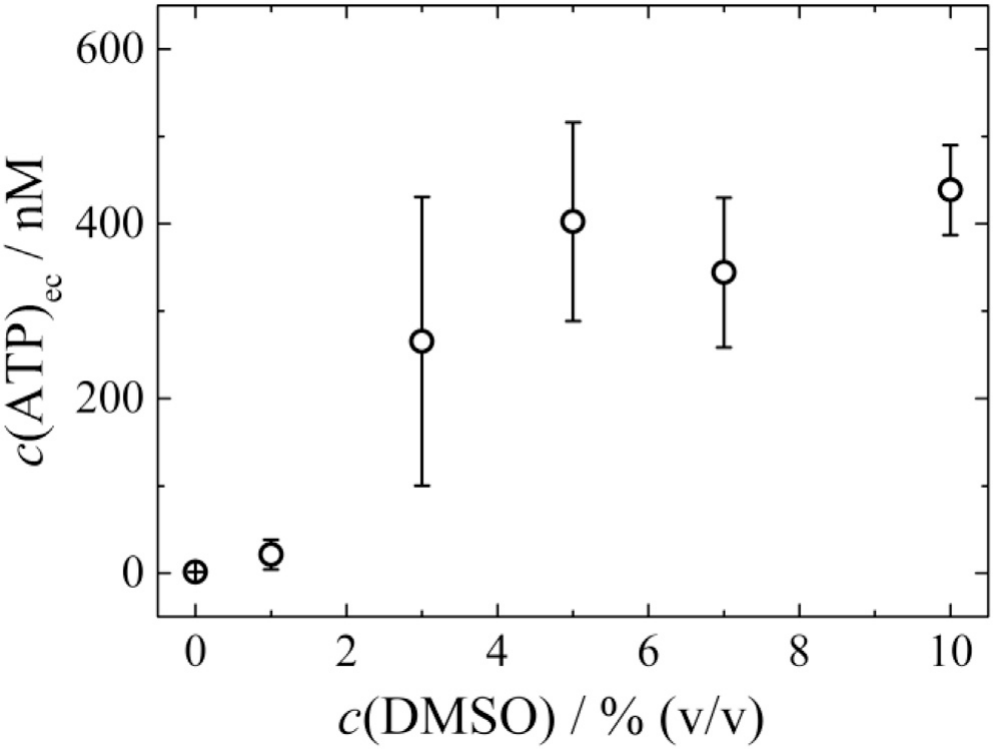
Extracellular levels of ATP in diluted blood samples incubated in buffers of varying DMSO concentration. Each point is an average of five measurements. The error bars denote the SD.

Fig. 10 shows a significant release of ATP from RBCs (elevated extracellular ATP levels) at DMSO concentrations as low as 3% v/v. A plateau is reached at around 5% v/v, which presumably indicates that the membrane is fully permeable to ATP above this concentration.

It would be important to clarify the mechanism by which DMSO could cause ATP release from red cells. However, transmembrane ATP transport in erythrocytes still remains unclear and a focus of continued debates. A growing body of evidence suggests that ATP transport across the erythrocyte membrane is regulated. Proteins implicated in the process include members of the family of the erythrocyte membrane ATP binding cassette, also involved in transmembrane ATP transport in other cell types (60). More recently, a signal transduction pathway for ATP release from red cells has been identified that includes the heterotrimeric Gi protein, adenylyl cyclase, protein kinase A, and the cystic fibrosis transmembrane conductance regulator (CFTR) (for a review, see (61)). Evidence for this pathway includes increased ATP release upon activation of Gi and, conversely, suppression of ATP release from cells when Gi is inhibited by pertussis toxin (61). Other studies found that pannexin 1 is a conduit for ATP release from red cells in response to low oxygen tension (62). A novel ATP release pathway from erythrocytes was proposed very recently, involving a complex between the translocase protein TSPO2, the adenine nucleotide transporter (ANT), and the voltage-dependent anion channel VDAC (63). Even though TSPO2, ANT, and VDAC are not abundant constituents of the erythrocyte membrane, it was argued that a high kinetic constant would make this pathway competitive (63). Yet, it is not clear which mechanism is dominant and under which conditions, and how the presence of DMSO might affect it. If we assume that ATP is released from red cells via a regulated transport, DMSO must be interfering with the release pathways. This is plausible because DMSO has been found to alter protein structure and function. For example, DMSO is reported to activate activator protein 1, which consequently stimulates the tumor suppressor protein HLJ1 (64). Another study found that even a low concentration of DMSO, up to 1.5% v/v, can alter protein secondary structure from *α*-helical to *β*-sheet (65). We could therefore hypothesize that DMSO might affect one or more constituents of the ATP transport pathway, thereby facilitating ATP release from the cell.

However, the notion of regulated transmembrane ATP transport in erythrocytes is still disputed. Sikora et al. (66) assert that the primary ATP release mechanism from red cells is hemolysis, i.e., ATP is released through structural disruptions in the plasma membrane and is not regulated. It is well known that DMSO is capable of increasing membrane permeability and could elicit changes in membrane organization, such as thinning and pore formation (see the Introduction). DMSO may cause membrane thinning, for example, through interactions with membrane cholesterol (65). Such structural disruptions would lower the energy barrier for transmembrane diffusion and may explain ATP release from erythrocytes in the presence of DMSO.

Clearly, further research is needed to clarify the effect of DMSO on regulated and unregulated transmembrane transport. Whatever the mechanism of DMSO-induced ATP release, these results could be of physiological importance, assuming DMSO also increases membrane permeability to ATP in other cell types. It is a common practice to use DMSO in relatively large concentrations (10%) as a cryoprotectant, for example for preservation (banking) of umbilical cord blood (67) rich in progenitor and stem cells (68). A decrease of cell ATP levels caused by DMSO may influence the cell viability and functionality upon thawing because a large number of cell functions and properties are dependent on ATP levels.

## Conclusions

Previous research suggested a degree of universality in the response of lipid bilayer membranes to DMSO, irrespective of their exact composition. A main motivation behind this study was to probe this notion further by investigating in detail common trends and differences in the response of lipid membranes to DMSO, using well-defined artificial bilayers of a different composition and bilayer phase state and a set of sensitive structural and mechanical techniques.

We established that two-component lipid membranes (POPC/ESM 1:1) existing in a phase-separated state at room temperature (a prevalent liquid disordered phase and a minority gel phase) are affected the most by DMSO. DMSO stabilized the gel phase by extending the thermal stability of this phase by ∼5°C. In contrast, DMSO has practically no effect on the thermotropic behavior of bilayers rich in cholesterol (POPC/ESM/cholesterol 1:1:3) residing in a liquid-ordered state.

Infrared structural studies revealed that DMSO reduces the available free volume of the two-component bilayers but has no effect on this parameter for the three-component system. Nevertheless, there are subtle differences between the two systems in the way DMSO modifies the available free volume in different membrane compartments, which could be attributed to the exact lipid composition and/or physical state of the membrane.

Experiments with GUVs reinforced some of the conclusions from the infrared studies. DMSO has a discernible effect on vesicles prepared from binary POPC/ESM 1:1 mixture by increasing the excess surface area of the vesicles (an effect previously termed “membrane loosening”), but we did not detect similar effects on ternary POPC/ESM/cholesterol 1:1:3 vesicles. DMSO had no effect on membrane bending rigidity of binary GUVs but induced an apparent stiffening of ternary GUVs*.* At present, the origin of this stiffening is unclear and may be due to DMSO-elicited structural alternations of the membrane region close to the lipid headgroups.

Compositional and organizational differences between artificial bilayers and native plasma membranes make it difficult to extrapolate directly between the two types of systems. Our single cell experiments on erythrocytes revealed a nonmonotonic effect of DMSO on the membrane bending rigidity: stiffening at low DMSO concentrations and softening at higher DMSO concentrations. Importantly, DMSO, even at relatively low concentrations, is capable of increasing the plasma membrane permeability to ATP. This effect may be significant in devising new cell cryopreservation strategies.

Taken in its entirety, this study illustrates that DMSO affects lipid membranes differently depending on their composition and/or physical state. Biological membranes are thought to be laterally inhomogeneous, consisting of microdomains of different composition and physical properties, e.g., lipid rafts suggested to be structurally based on the liquid-ordered phase (69,70). The actual picture is likely to be much more dynamic and complex, with membrane lateral inhomogeneities forming on different length and timescales. The situation is exacerbated by the fact that membranes have different biochemical and biophysical status, depending on their age, exposure to e.g., oxidative stress, disease status, etc. This means that DMSO is likely to have more complex differential effects on the heterogeneous membrane, depending on its local composition, structure, and dynamics.

## Author Contributions

B.G., Z.K., B.M., and B.-D.L. prepared the samples, conducted the experiments, and analyzed the results. J.M. wrote the software for analyzing membrane thermal fluctuations of GUVs. S.P., A.M., F.P., P.S., and P.G.P. conceived the idea for the project, designed the research, and coordinated the study. B.-D.L., B.M., and Z.K. contributed to the design of the research. B.G., P.S., and P.G.P. wrote the article. All authors reviewed the results, contributed to the analysis and interpretation, and reviewed, contributed to, and approved the final version of the manuscript.
